# The efficacy of nudge theory strategies in influencing adult dietary behaviour: a systematic review and meta-analysis

**DOI:** 10.1186/s12889-016-3272-x

**Published:** 2016-07-30

**Authors:** Anneliese Arno, Steve Thomas

**Affiliations:** Centre for Global Health, 7-9 Leinster Street South, Dublin 2, Ireland

**Keywords:** Nudge, Obesity, Cost-effective, Nutrition, Diet, Choice architecture

## Abstract

**Background:**

Obesity has become a world-wide epidemic and is spreading to countries with emerging economies. Previously tested interventions are often too costly to maintain in the long term. This leaves a need for improved strategies for management of the epidemic. Nudge Theory presents a new collection of methods, deemed “nudges”, which have the potential for low-cost and broad application to guide healthier lifestyle choices without the need for restrictive regulation. There has not yet been a large-scale examination of the effectiveness of nudges, despite several policy making bodies now considering their use.

**Methods:**

To address this gap in knowledge, an adapted systematic review methodology was used to collect and consolidate results from current Nudge papers and to determine whether Nudge strategies are successful in changing adults’ dietary choices for healthier ones.

**Results:**

It was found that nudges resulted in an average 15.3 % increase in healthier dietary or nutritional choices, as measured by a change in frequency of healthy choices or a change in overall caloric consumption. All of the included studies were from wealthy nations, with a particular emphasis on the United States with 31 of 42 included experiments.

**Conclusions:**

This analysis demonstrates Nudge holds promise as a public health strategy to combat obesity. More research is needed in varied settings, however, and future studies should aim to replicate previous results in more geographically and socioeconomically diverse countries.

**Electronic supplementary material:**

The online version of this article (doi:10.1186/s12889-016-3272-x) contains supplementary material, which is available to authorized users.

## Background

Despite widespread education and healthy eating campaigns, the prevalence of excessive body weight remains stubbornly high in many countries such as the United States, the United Kingdom, and Mexico while steadily climbing in others, including India and China [1].

From these climbing rates it would appear that most interventions previously attempted by public health policy are insufficiently effective. While there are several intensive interventions which have shown success in altering individuals’ body-mass indices (BMI) as well as their nutritional choices, these are largely short-term successes [2]. Moreover, they require massive time and monetary resources for each individual targeted. Many only function at a small scale, in isolated and easily tracked communities. Hence, these previously tested interventions are highly inefficient and would be too costly to implement at a national or population level [3]. Moreover, a less costly population-level intervention would enable under-resourced government bodies an affordable option, and encourage better health equity in the long term.

This situation demands that public health practitioners seek alternative strategies and interventions, in particular, those which could be applied at a population level and are a better value for public spending. From a public health standpoint, people are not generally making good decisions for their own health, or indeed for the health of society at large. It seems that individuals “irrationally” choose to ignore health warnings about obesity and recommendations for their nutritional choices, forming the basis of the obesity issue. Despite their irrational behaviour, predictable patterns may nonetheless be discernible when examined carefully. If research can reveal a method to use these patterns to encourage people to tend towards healthier options rather than the short-term pleasurable choices, a novel and powerful strategy to fight obesity may be established. Importantly, such a strategy would improve population health without coercion or restrictive legislation, instead using the sub-conscious processes present in all individual choices to gain a favourable outcome.

### Nudge theory

These assertions are the central tenets of Nudge Theory, a new collection of ideas put forth by Thaler and Sunstein in their 2008 book, Nudge: Improving decisions about Health, Wealth, and Happiness [4]. The authors contend that there exists a “choice architecture” which involves all of the outside forces that may subtly guide one’s decisions in one direction or another. Given the unavoidable existence of a choice architecture, the crucial next assumption is that a choice architect exists as well: a person or collection of persons who design the environment in order to make a certain option more likely to be chosen. For instance, items placed at eye-level in a supermarket may be selected more frequently than those near the floor. Another example might be manipulations to perception of serving sizes by using smaller serving utensils.

This benignly intentioned manipulation is labelled as “libertarian paternalism”, meant to improve the directions of people’s choices while maintaining freedom of choice. With this approach, they aim to reap the benefits of strong governmental oversight without the restrictive regulations and negative consequences. Nudge and libertarian paternalism are not necessarily new ideas, but the collation of these ideas within one umbrella term is a novel field. Consequently, not much work has been done in order to test the efficacy of adjustments in the choice architecture. Most of the previous work around these ideas has been speculative, and the field has been dominated by an ongoing debate about the morality of libertarian paternalism, which some consider to be an infringement of individual freedom of choice (e.g., [5, 6]). This focus on the ethics has left a gap in the literature as to whether or not nudge is an effective strategy for combatting obesity. Nudge previously has been examined in individual studies for its capacity to positively influence several other behaviours, including decreasing tobacco-use, increasing physical exercise, and encouraging financial planning, as described in [7]. However, it has not been systematically and quantitatively assessed for its effectiveness.

Much of the previous experimentation that would be classified as Nudge today was thus not originally published under this term, despite the fact that the interventions used would indeed fall under that category. Nevertheless, as governments have begun to use Nudge Theory strategies as a public health policy informer [7], it is essential to determine how effective a strategy it is. A previous systematic review [8] showed the majority of current Nudge literature is in the area of nutritional research, and as nutrition lies at the heart of one of the greatest health threats in the world – obesity – it seems a reasonable place to begin to answer a fundamental question: Does Nudge work, or does it not?

### Scope of research

In order to have a proper debate about whether to use these nudges, and particularly how to use them, it must first be understood whether or not they actually work. To do that, existing literature must be examined and the results compiled into an easily understood and communicated ‘bottom line’. Systematic reviews and meta-analyses have been established as an effective means of collecting and summarising current data and determining effectiveness of specific interventions for use in health care and policy [9]. Therefore, this research used this methodology to answer the research questions.

## Methods

### Research question

This review endeavoured to answer the following primary research question:

Is nudge theory an effective strategy in influencing adults aged 18 to 65 to change their dietary choices for healthier ones?

### Definition of terms

Healthier food was defined as more nutrient dense, lower calorie, lower salt, lower sugar, lower cholesterol, or lower fat. This definition was based on previous literature [10, 11]. More nutrient dense outcomes included consumption or purchase of more vegetables, fruits, whole grains, and other items reasonably identified and justified in the literature as healthy alternatives. Healthier outcomes also included decreased consumption of foods identified as “unhealthy,” such as snacks high in fat, salt, or sugar. Because healthier outcomes included both overall caloric consumption as well as nutrient dense food consumption, the terms ‘dietary’ and ‘nutritional’ are used interchangeably in this report.

### Inclusion and exclusion criteria

In addition to ensuring included studies were strictly responding to the research question, inclusion and exclusion criteria were also designed to exclude any study which demonstrated any risk of bias. These criteria are described in more detail in the corresponding sections below.

#### Population

In conjunction with the study question, only studies aimed at adults were used. Adulthood was defined by both legal and employment norms – i.e., a minimum of 18 years to a maximum 65 years of age. The age of 65 was selected as the upper limit because it is the age of retirement in many developed countries, while the lower limit of 18 is typical for the majority of legal recognitions. Studies with a range including above 65 (e.g., 22 to 75 years old) were included so long as the median age of participants remains below 65. In contrast, any study including children under 18 as well as adults was not included, unless the data was detailed in sub-group analysis allowing the researchers to only use adult data. Children under the age of 18 are the subjects of many studies, but these interventions would likely not be applicable to the general population as they would predominantly take place in schools. Finally, studies were not required to cover this entire age range, but were allowed to cover only a portion (e.g., 18–22 year olds).

Experiments were also required to include both men and women for the sake of general population value of the results. Numerous studies have noted that men and women respond differently to dietary interventions [12–14], and results obtained from only one gender may therefore be skewed. Indeed, many of the papers reviewed at the full-text and abstract level showed different results and levels of significance for the men and women included in the study. Studies using convenience sampling methods were not required to report the sample’s gender balance. For laboratory-based trials, however, included studies were not permitted to have a ratio higher than 70–30 in either direction.

Studies were excluded if participants were exclusively patients of a specific medical ailment, such as diabetes. Similarly, the subject pool could not be limited to obese or overweight people, as they would have well-documented differences in appetite, satiety, and restraint ratings [15]. Laboratory studies were required to exclude already-dieting participants, as they may have abnormal baseline eating patterns. Several studies included restraint ratings in their participant characteristics. Results for “restrained” and “unrestrained” eaters were pooled in some cases to remove this confounding effect.

In summary, included studies had to be aimed at a generic population, and not be primarily focused on a subset (e.g., women, diabetics, current dieters, etc.). Studies which reported sub-group analyses were not automatically excluded, provided they reported aggregated results or sufficient raw data to independently combine sub-group results in secondary analysis.

#### Study design

Studies must have been aimed at influencing behaviour relating to food consumption. Those focusing exclusively on beverages or alcohol consumption were not accepted. All study designs were accepted given they included a form of control or baseline comparator. For studies with parallel intervention groups, allocation of interventions had to be randomised, and baseline characteristics reported to ensure no significant pre-treatment group differences. Qualitative studies were excluded, though papers providing both qualitative and quantitative data were included so long as the quantitative data was reported according to the other standards described in this section.

#### Interventions (Comparisons)

Fundamentally, the intervention of interest must change the choice architecture while maintaining the autonomy of the test subject. Alterations to choice architecture include changes to the environment (e.g., olfactory or social), perception (e.g., emotional priming), availability of food (such as convenience or portion size), or knowledge-based changed (e.g., labelling). Any intervention which involved directly asking tests subjects about the experimental condition was excluded, as it would bias the participants. Similarly, interventions were not accepted which changed the fundamental properties of the food, such as energy density or fat content. Food appearance could be altered, however, such as a change in food unit size, colour, or odour. No options could be forbidden to the consumer or test subject, and no direct financial incentive to a participant could be involved.

Many of the reviewed and included studies examined several interventions simultaneously. For those examining both nudge and a non-nudge strategy, such as menu arrangement and pricing, respectively, results were required to be reported as a main effect of the nudge. If only the interaction effect was reported, the study was excluded.

#### Outcomes

Results had to be presented in terms of one of the following: calories, joules, grams, or purchases (either quantity purchased or a monetary amount). These had to be measured either directly or via purchase receipts; Participants were not permitted to report their own intake. Both absolute measurements and those reporting a change relative to baseline were accepted.

Studies which did not report a measurement of error – standard deviation, standard error, or confidence interval – were excluded. Studies that provided sufficient raw data for the independent secondary calculation of error, however, were included.

### Search strategy

The initial search strategy was based on a snowball method using the references from previously published similar systematic reviews, in particular [8]. Any other reviews identified during the preliminary search were also used to snowball articles, though these largely resulted in duplicates. These articles were reviewed at the title level to immediately identify those to be excluded. Those that were tentatively included were then reviewed at the abstract level, followed by the full text for those that continued to fit the criteria.

Following completion of screening of records retrieved via snowball, a systematic search of several databases was completed. The methodology for the search was adapted from [8]. The databases used were: EconLit, MEDLINE, PsycINFO, Embase, PubMed, and the Cochrane Library. As a final check point, Google Scholar was used to ensure the majority of literature had been screened.

Search terms included combinations, plurals, and various conjugations of the words relating to identified nudge strategies. The search strategy from [8] was used as a basis for search terms, but adjusted to reflect the more specific nutritional aim of this thesis. Studies published in the decade prior to the completion of our search phase (i.e., 2004–2014) were eligible for this review.

**Fig. 1:**
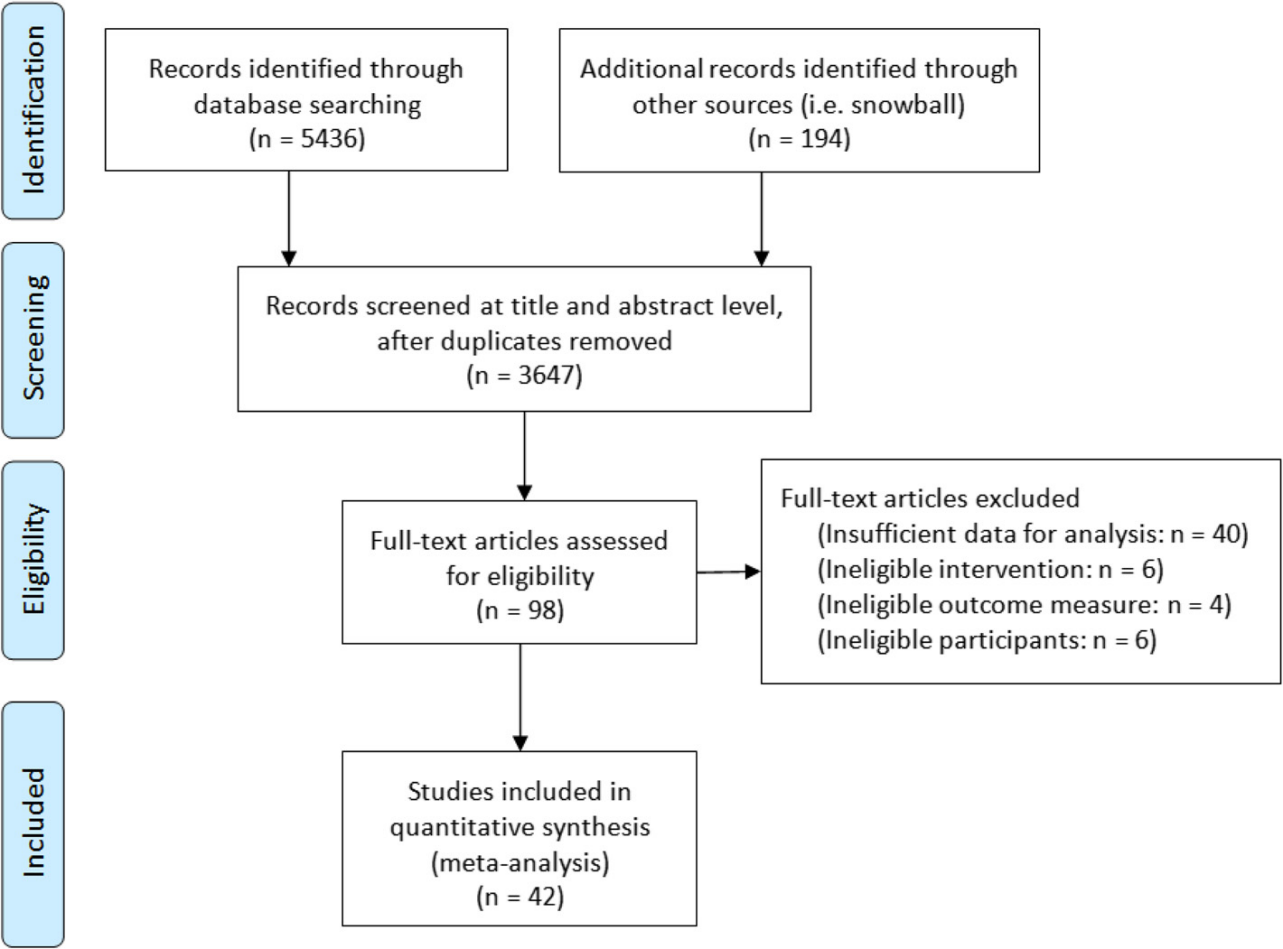
PRISMA flow diagram

Following retrieval of all records, duplicates were removed and records screened at the title level. Records were reviewed at title and abstract level simultaneously if this option was available via the database. Those not excluded due to key words in the titles were then reviewed at the abstract level. This was followed by a full text examination to finally determine inclusion or exclusion. Figure 1 summarises the search strategy and provides details of numbers of records at each stage.

All searching, screening, and data extraction was completed by author AA. Author ST approved the search and screening protocols, checked results of screening following full text review, and statistical results after data extraction and final calculations.

### Statistical analysis

In order to be able to compare heterogeneous outcomes, results were calculated as the absolute value percent change in frequency of a choice, or in consumption/purchases. These values were compiled into a forest plot and weighted according to their variance in order to demonstrate an overall trend in nudge intervention outcomes. The calculations necessary for the forest plot, including weighting of studies according to sample size and variance, were conducted using a previously published template [16]. This method of weighting results is in line with previously published reviews [9]. Statistical analyses were performed using Microsoft Excel 2010, while descriptive analyses of the included studies were performed using IBM SPSS v.21.

## Results

### Included study characteristics

This review identified 37 eligible papers [12, 17–52], with five included that reported results from several discrete experiments, resulting in 42 individual study results. Of these, 31, or 74 %, were randomised control trials (RCTs), two (5 %) were cohort studies, and nine (21 %) were cross-sectional studies. Laboratories were the most frequent study setting, accounting for 48 % of the included papers followed by canteens with 17 %. A notable majority of studies took place in the United States, and all were conducted in relatively wealthy Global North nations. Sample sizes used were most often below 100, with a further 31 % using between 100 and 500 participants. All of this information can be found in Table 1.

**Table 1:** Included studies characteristics

Author(s), Year	Study design	Sample Size	Setting	Country	Outcome [% change]^a^	Intervention (Choice Architecture alteration) description
Walsh and Kiviniemi, 2014 [17]	RCT	117	Laboratory	USA	28.3 ± 0.0343	Subjects’ affective associations with fruit and vegetables were experimentally altered using an implicit priming paradigm. They were then asked to choose between fruit or a granola bar for a snack.
Privitera and Zuraikat, 2014 [18]	RCT	56	Laboratory	USA	79.3 ± 17.7 [outlier]	Snacks were placed closer or farther away from subjects and their snack choices recorded and compared between control and experimental groups.
Gittelsohn et al., 2013 [19]	Cross-sectional (pre-post comparison)	145	Market	USA (Navajo)	7.84 ± 12.7	Availability of healthy options was increased over a 14-month period. Outcomes were measured in change of purchases of healthy items.
Kiesel and Villas-Boas, 2013 [20]	Cross-sectional (pre-post comparison)	4000	Market	USA	12.8 ± 17.2	Nutritional labelling was implemented over a four-week period. Outcomes were measured by purchases of healthy items.
van Kleef et al., 2012 [21]	RCT	67	Laboratory	USA	41.4 ± 11.8	Size of bowl used to serve snacks was varied and consumption measured and compared between control and experimental group.
Marchiori et al., 2012 [22]	RCT	88	Laboratory	Belgium	129 ± 0.703 [outlier]	Participants were given snacks in differently sized containers and their consumption measured.
Dumanovsky et al., 2011 [23]	Cross-sectional (pre-post comparison)	7311	Restaurant (fast-food)	USA	2.20 ± 5.02	Measurements of average energy content of purchases were made before and 21 months after addition of calorie labels to menu.
Finkelstein et al., 2011 [24]	Cross-sectional (pre-post comparison)	9823	Restaurant (fast-food)	USA	−1.31 ± 3.37	Calorie labelling was implemented in one county and compared to a county where it was not, using a five-month baseline period and six-month post-intervention period for results calculation.
Hoefkens et al., 2011 [25]	Cohort	657	Canteen	Belgium	2.20 ± 10.4	Nutritional information was posted in a workplace canteen and employees surveyed regarding their lunchtime choices pre- and post-intervention.
Ogawa et al., 2011 [26]	RCT	1684	Market	Japan	9.10 ± 1.26	Point-of-purchase nutritional information was added. Over a 60 day period, sales of healthy items were measured and compared between a control store and an experimental store.
Pulos and Leng, 2010 [27]	Cross-sectional (pre-post comparison)	16000	Restaurant (other)	USA	2.00 ± 1.09	Nutritional labelling was added in a restaurant. Entrée sales from 30 days before and 30 days after implementation were used for comparison.
Roberto et al., 2010 [28]	RCT	303	Restaurant (other)	USA	11.3 ± 5.34	Experimental subjects were given a label with calorie information while the control group was given a normal menu. Calorie intake was compared between the groups.
Shimizu et al., 2010 [29]	RCT	122	Laboratory	USA	21.7 ± 10.5	Presentational cues were used to indicate either a “meal” or a “snack” condition, and intake compared between control and experimental groups.
Wisdom et al., 2010 [30] - A	RCT	290	Restaurant (fast-food)	USA	5.80 ± 4.31	Calories were added to fast-food restaurant menus and average calorie intake per purchase compared between control and experimental groups.
Wisdom et al., 2010 [30] - B	RCT	342	Restaurant (fast-food)	USA	9.00 ± 7.64	In the experimental group, selection of unhealthy items was made less convenient by adding a step to the ordering process and moving them to the second page of the menu.
Chu et al., 2009 [31]	Cross-sectional (pre-post comparison)	13951	Canteen	USA	1.98 ± 0.14	Calorie information was posted in a workplace canteen. Calorie content of meals was calculated from sales data before and after calorie posting.
Gerend, 2009 [32]	RCT	288	Laboratory	USA	5.54 ± 5.52	In the experimental group, subjects received a menu with calorie information, while the control group received a regular menu. Calories per meal requested were compared between groups.
Kelly et al., 2009 [33]	RCT	43	Laboratory	UK (Northern Ireland)	11.7 ± 7.97	Portion size was randomly varied over four days in the lab and consumption measured and compared between conditions.
Stroebele et al., 2009 [34]	RCT	59	Laboratory	USA	44.8 ± 2.68	Subjects randomly received snack packs of different sizes for a week. Consumption measured and compared between groups.
Ueland et al., 2009 [35]	RCT	33	Laboratory	USA	7.17 ± 17.3	Subjects were told that they had received a standard portion or a larger portion, though in fact the amounts were the same. Consumption measured and compared between conditions.
Viskaal-van Dongen et al., 2009 [36]	RCT	51	Laboratory	Netherlands	36.8 ± 2.13	Foods with similar caloric content and either hidden or visible fat were presented and consumption measured and compared.
Bodor et al., 2008 [37]	Cross-sectional (internal comparison)	102	Neighbour-hood	USA	35.7 ± 1.73	Household survey regarding healthy food consumption. Groups were compared based on their proximity to a local grocery store.
Raynor and Wing, 2007 [38] - A	RCT	28	Home	USA	44.7 ± 2.68	Portion size adjusted and consumption measured and compared between groups.
Raynor and Wing, 2007 [38] - B	RCT	28	Home	USA	6.43 ± 32.2	Size of individual foot unit adjusted and consumption measured and compared between conditions.
Rolls et al., 2007 [39] (1)	RCT	23	Home	USA	14.6 ± 2.49	Portion size adjusted and consumption measured and compared between groups
Rolls et al., 2007 [40] (2)	RCT	119	Laboratory	USA	2.62 ± 1.85	Size of plate used was changed between control and experimental conditions, while the amount of food was held constant. Consumption was measured and compared between groups.
Antonuk and Block, 2006 [41]	RCT	67	Laboratory	USA	37.0 ± 18.2	Subjects were given snack food with randomly varying nutritional labelling. Their consumption was measured and compared.
Wansink et al., 2006 [42] - A	RCT	85	Laboratory	USA	12.6 ± 11.9	Larger serving utensils were used in the experimental group and consumption measured and compared.
Wansink et al., 2006 [42] - B	RCT	85	Laboratory	USA	23.6 ± 15.3	Larger bowls but identical serving sizes were used in the experimental group and consumption measured and compared.
Hetherington et al., 2006 [43] - A	RCT	37	Laboratory	UK (England)	11.2 ± 2.82	Subjects ate a meal either in the company of friends or of strangers. Consumption in the two conditions was compared.
Hetherington et al., 2006 [43] - B	RCT	37	Laboratory	UK (England)	15.4 ± 2.82	Subjects ate a meal either while watching TV or while alone. Consumption in the two conditions was compared.
Huang et al., 2006 [44]	RCT	456	Online	Australia	0.620 ± 0.165	Availability of healthier options in online supermarket was adjusted. Change in sales of healthy items was compared.
Norton et al., 2006 [12]	RCT	30	Laboratory	UK (England)	13.7 ± 4.02	Sandwiches were provided either with a variety of fillings or with a homogenous filling, though energy content was constant. Calorie intake was measured and compared.
Wansink and Kim, 2005 [45]	RCT	72	Movie Theatre	USA	31.2 ± 10.2	Popcorn in a movie theatre was sold in containers of various sizes. Consumption was measured and compared.
Wansink et al., 2005 [46]	RCT	54	Canteen	USA	42.2 ± 18.8	Experimental subjects were unknowingly given self-refilling bowls to make second helpings more convenient. Consumption was measured and compared.
Devitt and Mattes, 2004 [47]	RCT	20	Laboratory	USA	6.04 ± 5.70	Food unit size was randomly adjusted on four separate days. Consumption was measured and compared.
Diliberti et al., 2004 [48]	RCT	180	Canteen	USA	20.2 ± 0.567	Portion size of canteen entrées randomly varied in a workplace canteen. Sales data used to calculate energy content of meals.
Levitsky and Youn, 2004 [49]	RCT	13	Canteen	USA	23.6 ± 2.42	Portion size of canteen entrées randomly adjusted. Consumption measured and compared between conditions.
Rolls et al., 2004 [50] (1)	RCT	60	Laboratory	USA	12.1 ± 1.70	Subjects were given snacks of differing portion sizes. Consumption between control and experimental groups compared.
Rolls et al., 2004 [51] (2)	Cohort	75	Laboratory	USA	44.7 ± 1.90	Over four weeks, subjects were given sandwiches of varying sizes at a once weekly lab lunch. Consumption between conditions compared.
Steenhuis et al., 2004 [52] - A	Cross-sectional (pre-post comparison)	290	Canteen	Netherlands	−4.51 ± 8.92	In a workplace canteen, availability of healthy options was increased or held constant at seventeen worksites that were randomly assigned. Sales were used to calculate calorie content of meals and compared between conditions.
Steenhuis et al., 2004 [52] - B	Cross-sectional (pre-post comparison)	215	Canteen	Netherlands	−8.05 ± 9.98	At 17 randomly assigned worksites, healthy items were given additional labelling or left in original state. Sales were used to calculate calorie content of meals and compared between conditions.

^a^Negative values indicate behavioural changes opposite to those intended or desired (i.e., unhealthier)

### Meta-analysis

**Fig. 2:**
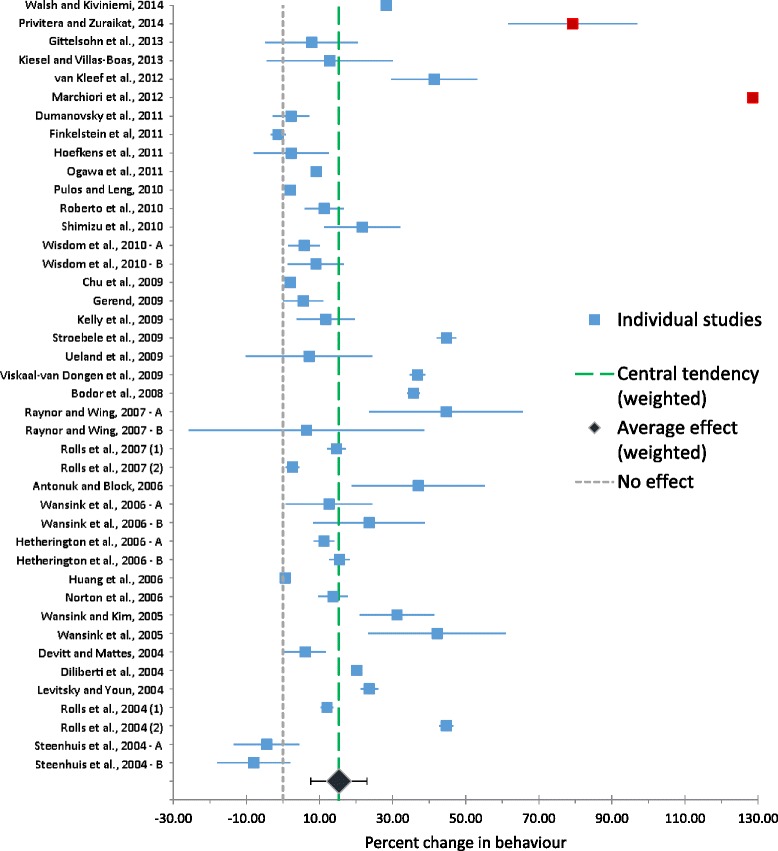
Forest plot of included studies’ results

When each study was weighted according to previously published methods [16], this analysis demonstrated that nudge interventions on average cause a 15.3 % (95 % confidence: 7.58, 23.0) increase in healthier consumption decisions, as measured by frequency of healthy choices or by overall intake. This result is shown in Fig. 2.

## Discussion

This analysis demonstrates that overall, nudge strategies successfully increase healthy nutritional choices. This review also demonstrated the current availability of evidence relating to Nudges and food policy, and highlighted the literature’s weaknesses. Our work built significantly upon the results of Hollands et al. (2013) by performing meta-analysis on the results of many studies initially identified in that work.

A significant limitation of this review is the lack of inter-rater reliability data due to the methods used, in which all studies were screened by a single reviewer with the second acting as a check point following completion of each screening stage. However, given the reasonable consistency of results across the articles it would suggest that this is not a major problem for the overall findings. Nevertheless, the validity of this work could have been improved by using a larger team for screening and reporting an inter-rater reliability metric such as Cohen’s Kappa.

As with any systematic review, this review and its results were limited by the presence of heterogeneous results and studies. Despite the presence of several sources of heterogeneity in this study’s included papers, discussed in the succeeding section, these are unlikely to have a large effect on the overall findings.

Firstly, the included studies differed in the precise population targeted. For example, a record retrieved during an initial trial search was aimed at a particular socioeconomic demographic by nature of its geographical setting. It is possible that interventions specifically designed for one demographic may not apply to another, or that compiling the results of such interventions could present heterogeneity to the results. However, by not excluding these studies, it was hoped to maintain the generalizability of these findings, as an entire population would include a plethora of socioeconomic statuses.

Similarly, that 100 % of the included studies were conducted in Global North nations, with a particular emphasis on the United States, may weaken the ability of the results of this review to be extrapolated to a more worldwide population, or to be applied in different national settings. This exclusive focus on relatively wealthy nations is indicative of an over-emphasis of obesity research on those nations; although this focus may be valid given the health trends of the past several decades, emerging economies are also poised to suffer the next wave of obesity and overweight, and this situation must be addressed.

A significant source of heterogeneity will likely arise, as in many past reviews, from the differences in how researchers measured their results. Results were evaluated as a proportional change in behaviour, outcome, or status, in order to be able to compile different results into one cohesive statement. While selection of this measure was necessary in order to be able to compile paper results and perform meta-analysis, it did necessitate the exclusion of some studies on the basis of lack of specific statistical data. Thus differences in measurement of outcomes not only presented heterogeneity of results calculated in this meta-analysis, but also shrank the pool of available data for statistical analysis. With more rigid reporting measures, the statistical results of this review could be updated to provide more robust results, such as effect size

Though nearly a third of the included papers reported a statistically insignificant result, there is always concern over publication bias [53], which would skew the results towards desirable and more statistically significant outcomes. Moving forward with Nudge Theory research, the field would benefit from reporting of all experimentation, whether its results are successful, unsuccessful, significant, or insignificant.

### Suggestions for future research

As described in the results section, included papers were exclusively from high-income countries (HICs) and from the United States in particular. Though these results provide a good theoretical framework for future work in other HICs, it is difficult to confidently ascertain the viability of these interventions in a low or middle-income country setting. Therefore, researchers should begin to conduct similar studies in more diverse country settings. By obtaining these results, the ability to extrapolate general policy themes from data will be strengthened.

While this review retrieved substantial data, our analyses would be strengthened by further availability of experiment results. The literature would benefit from repetition of nudges with demonstrated success at a broader, more population-based, level.

The research reported here focused on generally healthy male and female adults, as described in the methodology section and participant criteria. Due to the frequent reporting of different eating restraint scores between men and women [39, 45], it was chosen to focus only on mixed groups, but a worthwhile future venture would be to examine these same results as they are different between the sexes. It could be useful for policy makers to be aware of these discrepancies, and to examine the best intervention for each of them independently if nudges are to be considered in any gender-specific settings. At a minimum, such a review could confirm the results found for the general population for each group independently. These results could allow policy-makers to better tailor nudge approaches to specific settings.

Finally, a crucial step in future research will be to perform sub-group analyses on the results reported here and any additional studies that become available. These analyses could include differentiating results among different targeted populations to determine the best strategy for particular target groups, as mentioned above. They should certainly include a taxonomy of Nudge strategies and individual analysis for each of them, to determine if a particular method is better or worse than others. This would add benefit for health policy makers to be certain they are spending resources in the most effective ways.

## Conclusions

The results of this review and meta-analysis demonstrate that Nudge Theory strategies provide an effective and viable public health strategy in encouraging healthier eating choices in adults. As governments and policy-making bodies continue to consider the use of Nudge, it is essential they draw on strong evidence for its effectiveness. This work provides some of the first meta-analysis results to contribute to this required evidence base. It also clarifies some of the gaps in the current Nudge literature, highlighting where more research is needed to further strengthen the available evidence base.

## Abbreviation

HIC, high-income country

## Additional files

Additional file 1: Included studies. (DOCX 14 kb) Additional file 2: Full text exclusions. (DOCX 18 kb) Additional file 3: Search Strategies and yields. (DOCX 12 kb) Additional file 4: Data extraction. (XLSX 50 kb)
