# Supplementary material for: The efficacy of nudge theory strategies in influencing adult dietary behaviour: a systematic review and meta-analysis
Source: BMC Public Health. 2016 Jul 30;16:676. doi: 10.1186/s12889-016-3272-x (PMC4967524; doi:10.1186/s12889-016-3272-x)
Supplement: Additional file 1: — Included studies. (DOCX 14 kb) [file 12889_2016_3272_MOESM1_ESM.docx]

# **Included studies**

Antonuk, B. & Block, L.G., 2006. The Effect of Single Serving Versus Entire Package.

Bodor, J.N., Rose, D., Farley, T.A., Swalm, C. & Scott, S.K., 2008. Neighbourhood fruit and vegetable availability and consumption: the role of small food stores in an urban environment. Public health nutrition, 11, 413-420.

Chu, Y.H., Frongillo, E.A., Jones, S.J. & Kaye, G.L., 2009. Improving patrons' meal selections through the use of point-of-selection nutrition labels. American Journal of Public Health, 99, 2001.

Devitt, A.A. & Mattes, R.D., 2004. Effects of food unit size and energy density on intake in humans. Appetite, 42, 213-220.

Diliberti, N., Bordi, P.L., Conklin, M.T., Roe, L.S. & Rolls, B.J., 2004. Increased portion size leads to increased energy intake in a restaurant meal. Obesity research, 12, 562-568.

Dumanovsky, T., Huang, C.Y., Nonas, C.A., Matte, T.D., Bassett, M.T. & Silver, L.D., 2011. Changes in energy content of lunchtime purchases from fast food restaurants after introduction of calorie labelling: cross sectional customer surveys. BMJ, 343.

Finkelstein, E.A., Strombotne, K.L., Chan, N.L. & Krieger, J., 2011. Mandatory menu labeling in one fast-food chain in King County, Washington. American journal of preventive medicine, 40, 122-127.

Gerend, M.A., 2009. Does calorie information promote lower calorie fast food choices among college students? Journal of Adolescent Health, 44, 84-86.

Hetherington, M.M., Anderson, A.S., Norton, G.N. & Newson, L., 2006. Situational effects on meal intake: A comparison of eating alone and eating with others. Physiology & Behavior, 88, 498-505.

Hoefkens, C., Lachat, C., Kolsteren, P., Van Camp, J. & Verbeke, W., 2011. Posting point-of-purchase nutrition information in university canteens does not influence meal choice and nutrient intake. The American journal of clinical nutrition, 94, 562-570.

Huang, A., Barzi, F., Huxley, R., Denyer, G., Rohrlach, B., Jayne, K. & Neal, B., 2006. The effects on saturated fat purchases of providing internet shoppers with purchase-specific dietary advice: a randomised trial. PLoS clinical trials, 1, e22.

Kelly, M.T., Wallace, J.M., Robson, P.J., Rennie, K.L., Welch, R.W., Hannon-Fletcher, M.P., Brennan, S., Fletcher, A. & Livingstone, M., 2009. Increased portion size leads to a sustained increase in energy intake over 4 d in normal-weight and overweight men and women. British journal of nutrition, 102, 470-477.

Kiesel, K. & Villas-Boas, S.B., 2013. Can information costs affect consumer choice? Nutritional labels in a supermarket experiment. International Journal of Industrial Organization, 31, 153-163.

Levitsky, D.A. & Youn, T., 2004. The more food young adults are served, the more they overeat. The Journal of nutrition, 134, 2546-2549.

Marchiori, D., Corneille, O. & Klein, O., 2012. Container size influences snack food intake independently of portion size. Appetite, 58, 814-817.

Norton, G., Anderson, A. & Hetherington, M., 2006. Volume and variety: relative effects on food intake. Physiology & behavior, 87, 714-722.

Ogawa, Y., Tanabe, N., Honda, A., Azuma, T., Seki, N., Suzuki, T. & Suzuki, H., 2011. Point-of-purchase health information encourages customers to purchase vegetables: objective analysis by using a point-of-sales system. Environmental health and preventive medicine, 16, 239-246.

Privitera, G.J. & Zuraikat, F.M., 2014. Proximity of foods in a competitive food environment influences consumption of a low calorie and a high calorie food. Appetite, 76, 175-179.

Pulos, E. & Leng, K., 2010. Evaluation of a voluntary menu-labeling program in full-service restaurants. American Journal of Public Health, 100, 1035-1039.

Raynor, H.A. & Wing, R.R., 2007. Package unit size and amount of food: do both influence intake? Obesity, 15, 2311-2319.

Roberto, C.A., Larsen, P.D., Agnew, H., Baik, J. & Brownell, K.D., 2010. Evaluating the impact of menu labeling on food choices and intake. American Journal of Public Health, 100, 312.

Rolls, B.J., Roe, L.S., Halverson, K.H. & Meengs, J.S., 2007. Using a smaller plate did not reduce energy intake at meals. Appetite, 49, 652-660.

Rolls, B.J., Roe, L.S., Kral, T.V., Meengs, J.S. & Wall, D.E., 2004. Increasing the portion size of a packaged snack increases energy intake in men and women. Appetite, 42, 63-69.

Rolls, B.J., Roe, L.S. & Meengs, J.S., 2007. The effect of large portion sizes on energy intake is sustained for 11 days. Obesity, 15, 1535-1543.

Rolls, B.J., Roe, L.S., Meengs, J.S. & Wall, D.E., 2004. Increasing the portion size of a sandwich increases energy intake. Journal of the American Dietetic Association, 104, 367-372.

Shimizu, M., Payne, C.R. & Wansink, B., 2010. When snacks become meals: How hunger and environmental cues bias food intake. Int J Behav Nutr Phys Act, 7, 63.

Steenhuis, I., Van Assema, P., Van Breukelen, G., Glanz, K., Kok, G. & De Vries, H., 2004. The impact of educational and environmental interventions in Dutch worksite cafeterias. Health Promotion International, 19, 335-343.

Stroebele, N., Ogden, L.G. & Hill, J.O., 2009. Do calorie-controlled portion sizes of snacks reduce energy intake? Appetite, 52, 793-796.

Ueland, Ø., Cardello, A.V., Merrill, E.P. & Lesher, L.L., 2009. Effect of portion size information on food intake. Journal of the American Dietetic Association, 109, 124-127.

Van Kleef, E., Shimizu, M. & Wansink, B., 2012. Serving bowl selection biases the amount of food served. Journal of nutrition education and behavior, 44, 66-70.

Viskaal-Van Dongen, M., De Graaf, C., Siebelink, E. & Kok, F.J., 2009. Hidden fat facilitates passive overconsumption. The Journal of nutrition, 139, 394-399.

Walsh, E.M. & Kiviniemi, M.T., 2014. Changing how I feel about the food: experimentally manipulated affective associations with fruits change fruit choice behaviors. Journal of behavioral medicine, 37, 322-331.

Wansink, B. & Kim, J., 2005. Bad popcorn in big buckets: portion size can influence intake as much as taste. Journal of nutrition education and behavior, 37, 242-245.

Wansink, B., Painter, J.E. & North, J., 2005. Bottomless Bowls: Why Visual Cues of Portion Size May Influence Intake**. Obesity Research, 13, 93-100.

Wansink, B., Van Ittersum, K. & Painter, J.E., 2006. Ice cream illusions: bowls, spoons, and self-served portion sizes. American journal of preventive medicine, 31, 240-243.

Weijzen, P.L., 2008. Dynamics of food choice and sensory specific satiety: publisher not identified.

Wisdom, J., Downs, J.S. & Loewenstein, G., 2010. Promoting healthy choices: Information versus convenience. American Economic Journal: Applied Economics, 164-178.
