# Supplementary material for: The efficacy of nudge theory strategies in influencing adult dietary behaviour: a systematic review and meta-analysis
Source: BMC Public Health. 2016 Jul 30;16:676. doi: 10.1186/s12889-016-3272-x (PMC4967524; doi:10.1186/s12889-016-3272-x)
Supplement: Additional file 2: — Full text exclusions. (DOCX 18 kb) [file 12889_2016_3272_MOESM2_ESM.docx]

# Studies excluded at full-text stage

Bassett, M.T., Dumanovsky, T., Huang, C., Silver, L.D., Young, C., Nonas, C., Matte, T.D., Chideya, S. & Frieden, T.R., 2008. Purchasing behavior and calorie information at fast-food chains in New York City, 2007. American Journal of Public Health, 98, 1457.

Blass, E.M., Anderson, D.R., Kirkorian, H.L., Pempek, T.A., Price, I. & Koleini, M.F., 2006. On the road to obesity: Television viewing increases intake of high-density foods. Physiology & behavior, 88, 597-604.

Bollinger, B., Leslie, P. & Sorensen, A., 2010. Calorie posting in chain restaurants.

Borgmeier, I. & Westenhoefer, J., 2009. Impact of different food label formats on healthiness evaluation and food choice of consumers: a randomized-controlled study. BMC Public Health, 9, 184.

Breugelmans, E. & Campo, K., 2011. Effectiveness of in-store displays in a virtual store environment. Journal of retailing, 87, 75-89.

Burger, K.S., Fisher, J.O. & Johnson, S.L., 2011. Mechanisms behind the portion size effect: Visibility and bite size. Obesity, 19, 546-551.

Burton, S., Creyer, E.H., Kees, J. & Huggins, K., 2006. Attacking the obesity epidemic: the potential health benefits of providing nutrition information in restaurants. Journal Information, 96.

Coelho, J.S., Polivy, J., Peter Herman, C. & Pliner, P., 2009. Wake up and smell the cookies. Effects of olfactory food-cue exposure in restrained and unrestrained eaters. *Appetite,* 52**,** 517-520.

Dayan, E. & Bar-Hillel, M., 2011. Nudge to nobesity II: Menu positions influence food orders. Judgment and Decision Making, 6, 333-342.

De Castro, J.M., 2004. The time of day of food intake influences overall intake in humans. The Journal of nutrition, 134, 104-111.

Ebbeling, C.B., Garcia-Lago, E., Leidig, M.M., Seger-Shippee, L.G., Feldman, H.A. & Ludwig, D.S., 2007. Altering portion sizes and eating rate to attenuate gorging during a fast food meal: effects on energy intake. Pediatrics, 119, 869-875.

Elbel, B., Kersh, R., Brescoll, V.L. & Dixon, L.B., 2009. Calorie labeling and food choices: a first look at the effects on low-income people in New York City. Health Affairs, 28, w1110-w1121.

Engbers, L.H., Van Poppel, M.N., Paw, M.C.A. & Van Mechelen, W., 2006. The effects of a controlled worksite environmental intervention on determinants of dietary behavior and self-reported fruit, vegetable and fat intake. BMC Public Health, 6, 253.

Fiske, A. & Cullen, K.W., 2004. Effects of promotional materials on vending sales of low-fat items in teachers’ lounges. Journal of the American Dietetic Association, 104, 90-93.

Flood, J.E., Roe, L.S. & Rolls, B.J., 2006. The effect of increased beverage portion size on energy intake at a meal. Journal of the American Dietetic Association, 106, 1984-1990.

Freedman, M.R. & Connors, R., 2011. Point-of-purchase nutrition information influences food-purchasing behaviors of college students: a pilot study. Journal of the American Dietetic Association, 111, S42-S46.

Garg, N., Wansink, B. & Inman, J.J., 2007. The influence of incidental affect on consumers' food intake. Journal of Marketing, 71, 194-206.

Geier, A.B., Rozin, P. & Doros, G., 2006. Unit bias a new heuristic that helps explain the effect of portion size on food intake. Psychological Science, 17, 521-525.

Giesen, J.C., Payne, C.R., Havermans, R.C. & Jansen, A., 2011. Exploring how calorie information and taxes on high-calorie foods influence lunch decisions. The American journal of clinical nutrition, 93, 689-694.

Guéguen, N. & Petr, C., 2006. Odors and consumer behavior in a restaurant. International Journal of Hospitality Management, 25, 335-339.

Howlett, E.A., Burton, S., Bates, K. & Huggins, K., 2009. Coming to a restaurant near you? Potential consumer responses to nutrition information disclosure on menus. Journal of Consumer Research, 36, 494-503.

Kahn, B.E. & Wansink, B., 2004. The influence of assortment structure on perceived variety and consumption quantities. Journal of Consumer Research, 30, 519-533.

Koh, J. & Pliner, P., 2009. The effects of degree of acquaintance, plate size, and sharing on food intake. Appetite, 52, 595-602.

Lowe, M.R., Tappe, K.A., Butryn, M.L., Annunziato, R.A., Coletta, M.C., Ochner, C.N. & Rolls, B.J., 2010. An intervention study targeting energy and nutrient intake in worksite cafeterias. Eating behaviors, 11, 144-151.

Maas, J., De Ridder, D.T., De Vet, E. & De Wit, J.B., 2012. Do distant foods decrease intake? The effect of food accessibility on consumption. Psychology & health, 27, 59-73.

Madzharov, A.V. & Block, L.G., 2010. Effects of product unit image on consumption of snack foods. Journal of Consumer Psychology, 20, 398-409.

Marchiori, D., Corneille, O. & Klein, O., 2012. Container size influences snack food intake independently of portion size. Appetite, 58, 814-817.

Mishra, A., Mishra, H. & Masters, T.M., 2012. The influence of bite size on quantity of food consumed: a field study. Journal of Consumer Research, 38, 791-795.

Moray, J., Fu, A., Brill, K. & Mayoral, M.S., 2007. Viewing television while eating impairs the ability to accurately estimate total amount of food consumed. Bariatric Nursing and Surgical Patient Care, 2, 71-76.

Papies, E.K. & Hamstra, P., 2010. Goal priming and eating behavior: enhancing self-regulation by environmental cues. Health Psychology, 29, 384.

Pearson, T., Russell, J., Campbell, M.J. & Barker, M.E., 2005. Do ‘food deserts’ influence fruit and vegetable consumption?—A cross-sectional study. Appetite, 45, 195-197.

Peterson, S., Duncan, D.P., Null, D.B., Roth, S.L. & Gill, L., 2010. Positive changes in perceptions and selections of healthful foods by college students after a short-term point-of-selection intervention at a dining hall. Journal of American College Health, 58, 425-431.

Quartier, K., Christiaans, H. & Van Cleempoel, K., 2009. Retail design: lighting as an atmospheric tool, creating experiences which influence consumers’ mood and behaviour in commercial spaces.

Raynor, H.A., Van Walleghen, E.L., Niemeier, H., Butryn, M.L. & Wing, R.R., 2009. Do food provisions packaged in single-servings reduce energy intake at breakfast during a brief behavioral weight-loss intervention? Journal of the American Dietetic Association, 109, 1922-1925.

Roefs, A. & Jansen, A., 2004. The effect of information about fat content on food consumption in overweight/obese and lean people. Appetite, 43, 319-322.

Rolls, B.J., Roe, L.S. & Meengs, J.S., 2004. Salad and satiety: energy density and portion size of a first-course salad affect energy intake at lunch. Journal of the American Dietetic Association, 104, 1570-1576.

Rolls, B.J., Roe, L.S. & Meengs, J.S., 2006. Larger portion sizes lead to a sustained increase in energy intake over 2 days. Journal of the American Dietetic Association, 106, 543-549.

Rolls, B.J., Roe, L.S. & Meengs, J.S., 2010. Portion size can be used strategically to increase vegetable consumption in adults. The American journal of clinical nutrition, 91, 913-922.

Rozin, P., Scott, S., Dingley, M., Urbanek, J.K., Jiang, H. & Kaltenbach, M., 2011. Nudge to nobesity I: Minor changes in accessibility decrease food intake. Judgment and Decision Making, 6, 323-332.

Sacks, G., Rayner, M. & Swinburn, B., 2009. Impact of front-of-pack ‘traffic-light’nutrition labelling on consumer food purchases in the UK. Health promotion international, 24, 344-352.

Sacks, G., Tikellis, K., Millar, L. & Swinburn, B., 2011. Impact of ‘traffic‐light’nutrition information on online food purchases in Australia. Australian and New Zealand journal of public health, 35, 122-126.

Scott, M.L., Nowlis, S.M., Mandel, N. & Morales, A.C., 2008. The effects of reduced food size and package size on the consumption behavior of restrained and unrestrained eaters. Journal of Consumer Research, 35, 391-405.

Sharma, S., Wagle, A., Sucher, K. & Bugwadia, N., 2011. Impact of point of selection nutrition information on meal choices at a table-service restaurant. Journal of Foodservice Business Research, 14, 146-161.

Sigurdsson, V., Larsen, N.M. & Gunnarsson, D., 2011. An in-store experimental analysis of consumers' selection of fruits and vegetables. The Service Industries Journal, 31, 2587-2602.

Steenhuis, I., Kroeze, W., Vyth, E., Valk, S., Verbauwen, R. & Seidell, J., 2010. The effects of using a nutrition logo on consumption and product evaluation of a sweet pastry. Appetite, 55, 707-709.

Sutherland, L.A., Kaley, L.A. & Fischer, L., 2010. Guiding stars: the effect of a nutrition navigation program on consumer purchases at the supermarket. The American journal of clinical nutrition, 91, 1090S-1094S.

Temple, J.L., Johnson, K., Recupero, K. & Suders, H., 2010. Nutrition labels decrease energy intake in adults consuming lunch in the laboratory. Journal of the American Dietetic Association, 110, 1094-1097.

Thorndike, A.N., Sonnenberg, L., Riis, J., Barraclough, S. & Levy, D.E., 2012. A 2-phase labeling and choice architecture intervention to improve healthy food and beverage choices. American journal of public health, 102, 527-533.

Vadiveloo, M.K., Dixon, L.B. & Elbel, B., 2011. Consumer purchasing patterns in response to calorie labeling legislation in New York City. International Journal of Behavioral Nutrition and Physical Activity, 8, 51.

Van Herpen, E. & Trijp, H., 2011. Front-of-pack nutrition labels. Their effect on attention and choices when consumers have varying goals and time constraints. Appetite, 57, 148-160.

Vyth, E.L., Steenhuis, I.H., Heymans, M.W., Roodenburg, A.J., Brug, J. & Seidell, J.C., 2011. Influence of placement of a nutrition logo on cafeteria menu items on lunchtime food choices at Dutch work sites. Journal of the American Dietetic Association, 111, 131-136.

Vyth, E.L., Steenhuis, I.H., Vlot, J.A., Wulp, A., Hogenes, M.G., Looije, D.H., Brug, J. & Seidell, J.C., 2010. Actual use of a front-of-pack nutrition logo in the supermarket: consumers’ motives in food choice. Public health nutrition, 13, 1882-1889.

Wansink, B., 2010. From mindless eating to mindlessly eating better. Physiology & behavior, 100, 454-463.

Wansink, B. & Chandon, P., 2006. Can “low-fat” nutrition labels lead to obesity? Journal of marketing research, 43, 605-617.

Wansink, B. & Cheney, M.M., 2005. Super bowls: serving bowl size and food consumption. Jama, 293, 1723-1728.

Weijzen, P.L., Liem, D.G., Zandstra, E. & De Graaf, C., 2008. Sensory specific satiety and intake: The difference between nibble-and bar-size snacks. Appetite, 50, 435-442.
