# Supplementary material for: The efficacy of nudge theory strategies in influencing adult dietary behaviour: a systematic review and meta-analysis
Source: BMC Public Health. 2016 Jul 30;16:676. doi: 10.1186/s12889-016-3272-x (PMC4967524; doi:10.1186/s12889-016-3272-x)
Supplement: Additional file 3: — Search Strategies and yields. (DOCX 12 kb) [file 12889_2016_3272_MOESM3_ESM.docx]

# **Search Strategies**

## **EconLit**

TI ( nutri* or diet or habit* or avail* or convenience or environment* or smell* or scent* or food* or meal* or portion* or size* or container* or market or proxim* or knowledge* or label* or social or perception* or perceiv* or informat* ) AND TI ( nudg* or behav* or consum* or eat* or habit* or choice* or choice architecture or purchas* ) AND TI ( change* or alter* or intervention* or reduc* or increas* or less* or lower* or more* or option* or improve* or outcome* )

## **MEDLINE**

TI ( nutri* or diet or habit* or avail* or convenience or environment* or smell* or scent* or food* or meal* or portion* or size* or container* or market or proxim* or knowledge* or label* or social or perception* or perceiv* ) AND TI ( nudg* or behav* or consum* or eat* or habit* or choice* or choice architecture ) AND TI ( change* or alter* or intervention* or reduc* or increas* or less* or lower* or more* or option* or improve* or outcome* )

## **PsycINFO**

TI ( nutri* or diet or habit* or avail* or convenience or environment* or smell* or scent* or food* or meal* or portion* or size* or container* or market or proxim* or knowledge* or label* or social or perception* or perceiv* or informat* ) AND TI ( nudg* or behav* or consum* or eat* or habit* or choice* or choice architecture or purchas* ) AND TI ( change* or alter* or intervention* or reduc* or increas* or less* or lower* or more* or option* or improve* or outcome* )

## **Embase**

nutri* OR 'diet'/mj OR habit* OR avail* OR convenience OR environment* OR smell* OR scent* OR food* OR meal* OR portion* OR size* OR container* OR 'market'/mj OR proxim* OR knowledge* OR label* OR social OR perception* OR perceiv* OR informat* AND (nudg* OR behav* OR consum* OR eat* OR habit* OR choice* OR choice AND 'architecture'/mj OR purchas*) AND (change* OR alter* OR intervention* OR reduc* OR increas* OR less* OR lower* OR more* OR option* OR improve* OR outcome*) AND [article]/lim AND [adult]/lim AND [humans]/lim AND [2004-2014]/py

## **PubMed**

(TI ( nutri* or diet or habit* or avail* or convenience or environment* or smell* or scent* or food* or meal* or portion* or size* or container* or market or proxim* or knowledge* or label* or social or perception* or perceiv* or informat* ) AND TI ( nudg* or behav* or consum* or eat* or habit* or choice* or choice architecture or purchas* ) AND TI ( change* or alter* or intervention* or reduc* or increas* or less* or lower* or more* or option* or improve* or outcome* ))

## **Cochrane**

(nutri* or diet or habit* or avail* or convenience or environment* or smell* or scent* or food* or meal* or portion* or size* or container* or market or proxim* or knowledge* or label* or social or perception* or perceiv* or informat*) and (nudg* or behav* or consum* or eat* or habit* or choice* or choice architecture or purchas*) and (change* or alter* or intervention* or reduc* or increas* or less* or lower* or more* or option* or improve* or outcome*):ti,ab,kw

# **Search dates and yields**

| **Database** | **Date** | **Yield** |
| --- | --- | --- |
| EconLit | 1 June 2014 | 255 |
| MEDLINE | 1 June 2014 | 890 |
| PSYCInfo | 1 June 2014 | 771 |
| Embase | 30 May 2014 | 1,555 |
| PubMed | 2 June 2014 | 619 |
| Cochrane | 2 June 2014 | 1,346 |
